# The Uptake of Sporopollenin Exine Capsules and Associated Bioavailability of Adsorbed Oestradiol in Selected Aquatic Invertebrates

**DOI:** 10.1007/s00128-021-03364-8

**Published:** 2021-08-30

**Authors:** Emma Chapman, Aimilia Meichanetzoglou, Andrew N. Boa, Hanne Hetjens, Sonja Faetsch, Johnny Teuchies, Sebastian Höss, Dean Moore, Lieven Bervoets, Paul Kay, Susanne Heise, Paul Walker, Jeanette M. Rotchell

**Affiliations:** 1 Department of Biological and Marine Sciences, University of Hull, Cottingham Rd, Hull, HU6 7RX UK; 2 Department of Chemistry, University of Hull, Cottingham Road, Hull, HU6 7RX UK; 3 Department of Biology, SPHERE, University of Antwerp, Groenenborgerlaan 171, 2020 Antwerp, Belgium; 4 Hamburg University of Applied Sciences, Ulmenliet 20, 21033 Hamburg, Germany; 5 Ecossa, Giselastr. 6, 82319 Starnberg, Germany; 6 School of Geography/water@leeds, University of Leeds, Leeds, LS2 9JT UK; 7 SOCOTEC UK Ltd, Etwall House, Bretby Business Pk, Ashby Road, Burton on Trent, DE15 0YZ UK

**Keywords:** Sporopollenin, Oestrogen, Uptake, Bioavailability, Gene expression

## Abstract

*Lycopodium clavatum* sporopollenin exine capsules (SpECs) are known to both adsorb and absorb chemicals. The aim of the present work was to determine whether oestradiol (E2) is ‘bioavailable’ to bioindicator species, either pre-adsorbed to, or in the presence of, SpECs. SpEC uptake was confirmed for *Daphnia magna* and *Dreissena bugensis*. E2 levels varied among treatments for *Caenorhabditis elegans* though there was no relationship to SpEC load. E2 was not detected in *D. bugensis* tissues. Expression changes of general stress and E2-specific genes were measured. For *C. elegans*, *NHR-14* expression suggested that SpECs modulate E2 impacts, but not general health responses. For *D. magna*, SpECs alone and with E2 changed *Vtg1* and general stress responses. For *D. bugensis*, SpECS were taken up but no E2 or change in gene expression was detected after exposure to E2 and/or SpECs. The present study is the first to investigate SpECs and bound chemical dynamics.

Oestrogens enter the aquatic environment mainly via sewage treatment works (STWs) effluents. The most commonly found are the natural compounds, E1, E2 and E3 followed by synthetic EE2, none of which are significantly removed during STW clean-up processes (Racz and Goel 2010). Using the current UK population as an example, it has ~ 38 million adults (not including the elderly), amounting to ~ 167 kg of oestrogens arriving at STWs each year. A further 500 kg of consumed prescribed oestrogens each year can be added according to calculations by Stuer-Lauridsen and Kjolholt (2000). Such environmental oestrogens are deemed to pose a human and ecological risk at levels over 0.028 and 0.035 ng L^−1^ respectively, with links to elevated risk of cancer, cardiovascular disease and detrimental reproductive effects in humans (Adeel et al. 2017).

Pollens and spore grains are part of the plant reproductive system, with a double layered wall structure and exine layer, a resistant organic polymer, sporopollenin, of debated structure (Li et al. 2019; Mikhael et al. 2020). Sporopollenin can be extracted to provide empty shells, called sporopollenin exine capsules (SpECs). The surface of SpECs are penetrated by channels, giving a high surface area and absorption capacity (Thio et al. 2011; Rowley et al. 2011), thus potentially useful in removing chemicals at STWs. Here, we aimed to determine whether E2 is ‘bioavailable’ to a variety of bioindicator species from freshwater environments, once adsorbed to or in the presence of *Lycopodium clavatum* SpECs. The effects of E2 on aquatic invertebrates, quantified via LCMS-MS and gene expression, were investigated in E2-dosed water contrasting with E2-loaded SpECs to explore the potential application of SpECs as E2 adsorbents in wastewater treatment.

## Materials and Methods

SpECs were extracted from spores of *L. clavatum* using 9 M HCl. SpECs (3 g) were added to a solution of E2 in ethanol (0.1 μg/mL, 30 mL). The solvent was evaporated, then the solids resuspended in 10 mL ethanol, rinsing well the flask walls. Evaporation to dryness gave the E2-loaded SpECs (1 ng E2/mg). For exposure experiments, selection SpECs mass for a given volume gave, in theory, the concentrations of E2 if it all dissolved. *Caenorhabditis elegans* (Rhabditida, Rhabditidae) nematodes (n =  ~ 400 individuals, three replicates), *Daphnia magna* (Cladocera, Daphniidae) planktonic crustaceans (n = 25 in three replicates) and *Dreissena bugensis* (Myida, Dreissenidae) quagga mussels (n = 6 in three replicates) were exposed to the following treatment groups: (i) control; (ii) E2 10 ng L^−1^; (iii) E2 100 ng L^−1^; (iv) untreated SpECs; (v) E2-loaded SpECs. The masses of E2-loaded SpECs were chosen in each case to reflect the potential maximal concentration of E2 if it was fully desorbed (bioavailable) from the SpECs, and matched the E2 concentrations at low (10 ng L^−1^) and high dose (100 ng L^−1^) exposures. The same masses of untreated SpECs were also used. Exposure volumes used were 50 mL for *C. elegans* and *D. magna* and 500 mL for *D. bugensis*. Exposure duration, without feeding, was 24 h for *C. elegans* (at 20°C), 48 h for *D. magna* (20 ± 1°C, 16:8 light–dark cycle) and for 72 h for *D. bugensis* (15 ± 1°C, 16:8 light–dark cycle). For *C. elegans*, all exposure solutions or suspensions were prepared in M9-medium (pH = 7.2). Exposure solutions were prepared with Millipore ultrapure water for *D. magna* and dechlorinated tap water for *D. bugensis*, pH = 7.8. On termination, one third of the *D. magna* and *D. bugensis* were fixed in 4% formaldehyde, one third (for all species) immediately frozen at − 80°C for chemical analysis, and one third (of all species) stored in RNALater at − 20°C for gene expression analysis. *C. elegans* was not analysed for SpEC body burdens because it is unable to ingest particles > 3 µm (Fueser et al. 2019).

SpEC numbers in *D. magna* gut were counted and photographed under visible light and with a CY3 filter (orange) on an Olympus IX71 inverted microscope using CellSens Entry software (Olympus, UK). Statistical analysis (Kruskal–Wallis Test (*KW*) n = 12–21, followed by Dunn’s comparisons with control group) was carried out in GraphPad InStat v3. *D. bugensis*, stored in 4% formaldehyde, were halved, washed, dehydrated, cleared, and embedded in paraffin wax. Sections (10 μm) were dried overnight, cleared with Histoclear II and mounted with DPX medium. Three fields of view of each section were photographed and SpECs were counted. *C. elegans* (n = 3 pools of animals stored in ethanol of 600 μL volume each, centrifuged and ethanol removal by pipetting/evaporation) and *D. bugensis* (n = 5–6 individuals from each exposure; 1.89 ± 0.4 g wet weight), were pooled, homogenised and freeze-dried; (0.2 ± 0.06 g dry weight). QuEChERS extractions were performed on the pooled *C. elegans* individuals and *D. bugensis* tissues. All samples were spiked with 100 ng 17β-estradiol-2,3,4-^13^C3 internal standard (Sigma Aldrich, UK) in acetonitrile prior to extraction using QuEChERS (Phenomenex, UK). Samples were reconstituted with 300 µL 100 μg mL^−1^ 4-(dimethylamino)benzoyl chloride (99% purity) (Sigma Aldrich, UK) prepared in dry acetone with a 4-(dimethylamino)pyridine catalyst, and derivatised at 60°C for 1 h, evaporated to dryness under nitrogen and reconstituted in 1 mL acetonitrile. These were filtered (0.22 μm) and stored at − 20°C. E2 analysis was conducted on a Shimadzu LCMS-8060 Triple Quadrupole Mass Spectrometer in positive ESI–MS/MS mode with quantification/qualifier MRMs at m/z 420–148/420–166 (DMABC-E2) and 423–148/423–166 (DMABC-^13^CE2). The calibration curve was in triplicate from 0.01 to 10 ng μL^−1^ and calibration coefficients (*R*^2^) were 0.998 and 0.999.

For gene expression analysis,total RNA extraction was performed on *C. elegans* and *D. magna* pooled from each experimental treatment. For *C. elegans*, this comprised n = 3 pools of pelleted individuals (n =  ~ 400 per pool) from each exposure group. For *D. magna*, 14–28 individuals from each exposure group comprised one single pooled sample. *D. bugensis*, extraction was performed on ~ 10 mg of tissue from each experimental treatment. RNA was extracted with the High Pure RNA Tissue Kit (Roche, UK). *C. elegans* and *D. magna* had a proteinase K (20 μL 600 U mL^−1^, Thermo Scientific) and triton X-100 (1 μL) incubation at 37°C for 15 min, followed by snap freezing at − 80°C for 10 min. After defrosting, the lysate was sheared through a 20-gauge needle five times before sonication. cDNA synthesis was performed using 170 ng mussel RNA and 120 ng of each *C. elegans*/*D. magna* RNA pool with the Precision Nanoscript2 Reverse Transcription Kit with random primers (PrimerDesign, UK).

qPCR assays were optimised for *NHR-14* (nuclear hormone receptor 14), *CAT* (*catalase)*, and reference genes *pmp-3* (peroxisomal membrane protein) and *cdc-42* (cell division control protein 42 homolog) for *C. elegan*s. *Vitellogenin 1* (*Vtg1)* and superoxide dismutase (*SOD)*, and reference gene ubiquitin-conjugating enzyme (*UBC*), were optimised for *D. magna*. For mussel, an *ER* product was obtained with primers 5′GATTATGTTTGTCCAGCTAC3′ and 5′TTGTCAGGGTGGTATTTCTG3′ based on *Mytilus edulis* Me*ER2* (GenBank **AB257133.2**). *ER, CAT* and reference genes *18S*, *28S* and *16S* were optimised for *D. bugensis*. qPCR reactions contained 10 μL PrecisionPLUS 2 × qPCR MasterMix with SYBR Green for the ICycler (PrimerDesign, UK) 7 µL molecular-grade water, 2 µL primer mix and 1 µL cDNA (from ~ 120 ng RNA). Reactions were in duplicate on a CFX96 Real Time PCR Detection System (Bio-Rad, UK), with cycling conditions: 95°C for 2 min, 40 cycles of 95°C for 10 s, 60°C for 1 min and 72°C for 1 min. Reaction efficiencies were 90%–110% (Bustin et al. 2009). Relative mRNA expression levels were assessed using the 2^−ΔCT^ version of the comparative CT method with the geometric mean of the reference genes (Livak and Schmittgen 2001) and statistically analysed in SPSS v24 (*KW* test, n = 3–5).

## Results and Discussion

**Fig. 1:**
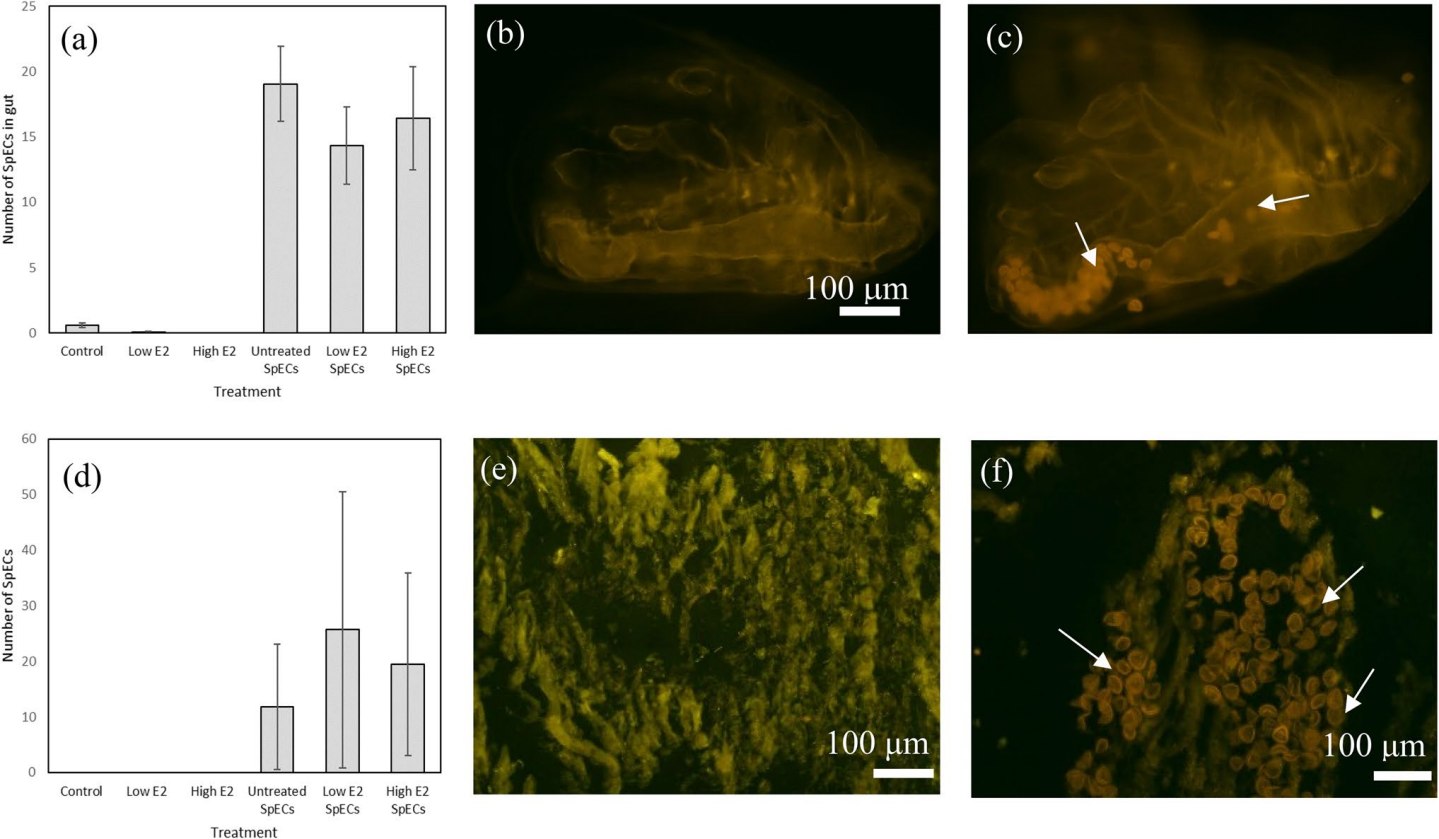
Mean number of SpECs counted in **a** *D. magna* gut (n = 12–21) and (**d**) randomly selected sections from *D. bugensis* whole body tissues (n = 6) from each treatment ± SEM; and micrographs under CY3 fluorescence filter showing unstained whole *D. magna* and sections of *D. bugensis* from control (**b**, **e**) and SpECs-exposed conditions (**c**, **f**). Arrows indicate SpECs

SpECs were detected in the gut of *D. magna* from SpECs-exposed treatments (Fig. 1b, c) (mean ± SEM): Untreated SpECs (19.05 ± 2.86), Low E2 with SpECs (14.33 ± 2.96) and High E2 with SpECs (16.42 ± 3.93) (Fig. 1a) and were significantly higher than in the control group (*KW* = 91.45, df = 5, *p* < 0.001, Dunn’s test *p* < 0.001). The mean number of SpECs in non-SpECs exposed *D. magna* treatments was < 1 and did not significantly differ from controls (*p* > 0.05) (Fig. 1a): Control (0.57 ± 0.18), Low E2 (0.05 ± 0.05) and High E2 (0). SpECs were also observed in the soft tissues of *D. bugensis* (Fig. 1d, f), but not in mussels exposed to control or E2 alone (Fig. 1e). A high degree of individual variation, in the randomly selected field of vision tissue sections, across the SpEC exposure groups, was detected (Fig. 1d).

**Fig. 2:**
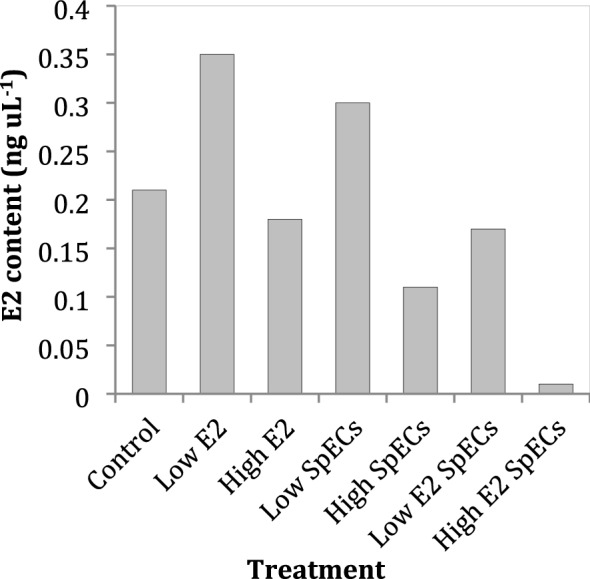
Oestradiol content in pooled *C. elegans* samples (n =  ~ 400 per pool)

The E2 analysis LOQ (peak signal to noise 10:1) was 0.02 ng μL^−1^ and LOD (peak signal to noise 3:1) was 0.01 ng μL^−1^. The recovery and derivatisation efficiency for labelled E2 varied widely between 0 and 49% due to the requirement of detecting derivatised E2 at low concentrations. For those samples at 0%, or close to 0%, the results were rejected and discarded. E2 levels in all *C. elegans* pooled samples for all exposure groups were 0.19 ± 0.11 ng μL^−1^ (mean and SD), where animals exposed to Low E2 (10 ng μL^−1^) were increased, yet SpECs loaded with High E2 (100 ng μL^−1^) were decreased, relative to controls (Fig. 2), though significance between groups is not quantifiable having used single pooled samples. E2 was not detected in any *D. bugensis* tissues.

**Fig. 3:**
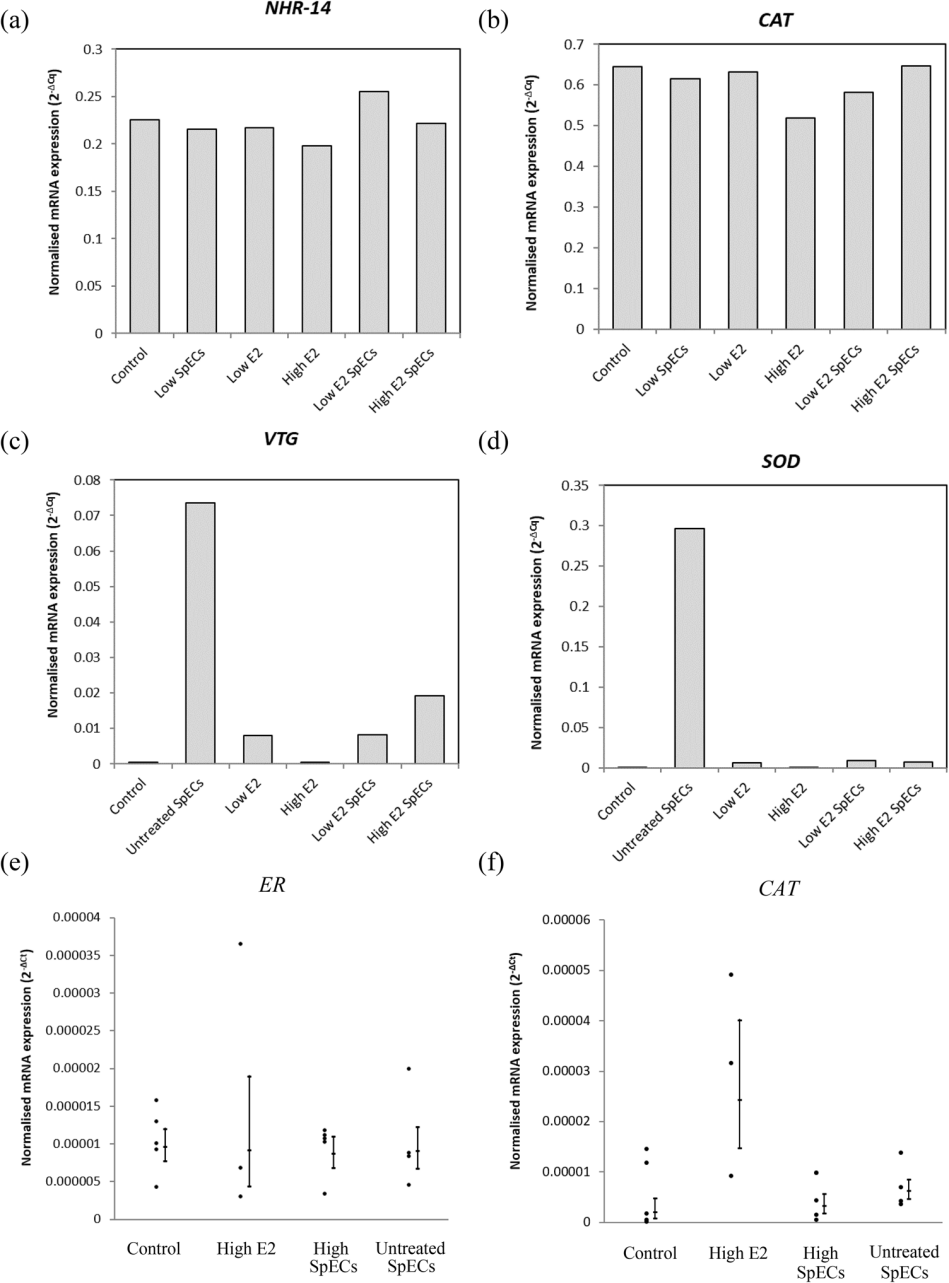
mRNA expression (arbitrary units) of **a** *NHR-14* and **b** *CAT* in *C. elegans* (n = 1200), **c** *VTG1* and **d** *SOD* in *D. magna* (n = 14 or 28), and **e** *ER* and **f** *CAT* in *D. bugensis* (n = 3–5, mean ± SEM)

mRNA expression of *NHR-14* and *CAT* were investigated in single pools of *C. elegans* from: control, SpECs Low, SpECs High, Low E2, High E2, SpECs + Low E2, SpECs + High E2. Expression of *NHR-14* in the treatments did not exceed that of the control group, with the exception of the SpECs + Low E2 treatment (Fig. 3a). *CAT* expression was lower in treatments compared to control with the exception of the SpECs + High E2 treatment (Fig. 3b). Expression of *Vtg1* and *SOD* were investigated in pooled samples of *D. magna* from the following conditions: Control, Untreated SpECs, Low E2, SpECs + Low E2, High E2, and SpECs + High E2 (Fig. 3c,d). Exposure to SpECs, either untreated (for *SOD*) or with E2 bound (*Vtg1* and *SOD*), resulted in an increase in expression relative to the control or E2 alone groups. Expression of *ER* and *CAT* were investigated in *D. bugensis* tissues from: control, High E2, SpECs + High E2, and Untreated SpECs. No significant differences in expression were found between groups for either gene; *ER* (*KW* = 0.493, df = 3, *p* = 0.92) and *CAT* (*KW* = 4.35, df = 3, *p* = 0.226) (Fig. 3e, f).

Histological analysis confirms the uptake of SpECs in *D. magna* and *D. bugensis* with significant numbers observed in the gut and other tissues, respectively for each species. Even if SpECs adsorb E2 to the extent that it is no longer bioavailable, their removal from wastewater before release into a receiving environment, such as a river beside a STW outfall source, may still be required due to the wider implications of potential uptake by organisms. For example, as exines are durable structures, they are resistant to degradation and digestion, so they will persist in the environment. The nutritional content of ingested grains is derived from the encapsulated cytoplasm, and this plus the digestibility of pollen grains varies among plant species and consumers (Roulston and Cane 2000). An exclusive diet of pollen was found to negatively affect development of zooplankton including *D. longispina*; whereby the presence of microorganisms is required to degrade them to an intermediate trophic level (Masclaux et al. 2011). As SpECs are empty capsules, they lack the nutritional component of intact grains and have been proposed as a dilution agent in artificial diets for arthropod larvae (Tainsh et al. 2020). The effects of dietary dilution by SpECs on invertebrates are subject to further investigation.

Having confirmed uptake of SpECs by two organisms, it is important to know whether any chemicals bound to the surface are bioavailable. Chemical analysis confirmed that the E2 levels in *C. elegans* samples varied between treatments, suggesting that E2 is present and at varying levels according to treatment group, though no relationship between SpEC and E2 loading values was observed. In contrast to *C. elegans*, no E2 was detected in any *D. bugensis*, tissues. *C. elegans* and *D. bugensis* are both invertebrates and the presence and functional role of vertebrate steroids is debated (Scott, 2012).

The variation of E2 tissue levels observed in the *C. elegans* across the treatment groups, resulted in an apparent increase in E2 in *C. elegans* exposed to low levels (but not high levels) of E2 alone, suggesting that such levels are internally regulated by these organisms. Furthermore, the exposure treatment using high levels of E2 bound to SpECs resulted in a decrease in tissue E2 levels, suggesting that the SpEC loading may have a protective effect and/or prevented E2 uptake, relative to E2 alone.

In contrast to the variable E2 levels detected in *C. elegans*, no E2 was detected in any of the *D. bugensis* tissues in any treatment group. For molluscan species, the evidence for modulated gene expression of the related oestradiol receptor (ER) and associated vitellogenin (VTG) egg yolk protein is debated. Some studies confirm presence and up-regulation of oestrogen-responsive genes, or increased levels VTG proteins, following controlled E2 exposure (Leonard et al. 2017) and others conclude that the receptor is non-constitutive in molluscs and other invertebrates (Thornton et al. 2003), having no functional, or at least, no reactive role in oestrogen chemical responses in such organisms (Scott, 2012). The levels of E2 in bivalves have been shown to vary throughout the year; the profile is synchronised with variations of oocyte diameter and gonad index (Osada et al. 2004). Subsequently, E2 is considered to exhibit a seasonal change associated with the reproductive cycle and to be involved in the regulation of several reproductive processes in bivalves such as vitellogenesis (Osada et al. 2004). On the other hand, possible explanations for the lack of induction in bivalves have been suggested (Thornton et al. 2003), including the rapid esterification of oestrogens to conjugates (Labadie et al. 2007), which may also explain the lack of oestrogens detected in the tissues. The role of oestrogens and their functional mechanism of action in bivalves are therefore far from clear, with the results obtained using *D. bugensis* seemingly further underlining this complexity. More exposures are required to elucidate the role that SpECs might play as a vector for adsorbed chemicals, using a chemical contaminant for which the uptake measurement is simple (avoiding recovery problems and derivatisation steps) and related to easily detected biological endpoints. This work represents a first attempt at monitoring the associated impacts of contaminants bound to SpECs and offers important insights into how to proceed in future studies.

The biological endpoints adopted herein relied on invertebrate species having an E2 specific and also general stress response to SpEC/E2 exposure. Previous work conducted by Mimoto et al. (2007), using *C. elegans* in controlled laboratory exposures to oestrogenic chemicals, reported that expression of the *NHR-14* can be up-regulated by oestrogens including E2. For these *C. elegans*, expression of *NHR-14* in the SpECs + Low E2 treatment (Fig. 3) was the only exposure group to show E2 specific modulation of the gene. *CAT* mRNA expression, the general stress indicator, differed in the exposed treatment groups compared to the controls with the exception of the SpECs + High E2 treatment. These varying expressions suggest that E2 alone and E2 bound to SpECs do have differing biological impacts, but the pattern is unclear and further experiments, with more replicates is required.

Using daphnia for uptake and gene expression analysis is not a new approach (Heckmann et al. 2006), yet the mechanisms involved in the response to oestrogen exposure are not known, though the egg yolk protein vitellin has been shown to be induced by exposure (albeit at high levels of 1000 μg L^−1^) of EE2 (Clubbs and Brooks 2007). For both genes, exposure to SpECs, either untreated or with E2 bound, gave an increase in expression relative to the control or E2 alone groups (Fig. 3), highlighting the SpECs themselves, rather than E2, as the possible cause. The E2-specific endpoint *Vtg1* failed to indicate whether E2 was available or not in each exposure scenario. Daphnia *Vtg1* is also known to be elevated after thermal stress (Samanta et al. 2020), and inhibited by exposure to perfluoroethylcyclohexane sulfonate (Houde et al. 2016) or bisphenol A (Chen et al. 2021). In addition to *Vtg1*, Daphnia also possess another vitellogenin gene *Vtg2* (Tokishita et al. 2006) but, similarly to these results presented, does not appear to respond to oestrogenic chemical activity (Hannas et al. 2011). More recently, the transcriptomic response of *D. magna* to E2 has been investigated revealing differentially-expressed genes with functions in immune responses, disease resistance, cancer-related functions and metabolism (Zheng et al. 2020). Such genes may provide alternative biomarkers for daphnia E2 exposure in future.

Gene expression studies are dependent on the extraction of high-quality template (Bustin et al. 2009), however this can be challenging when samples are small and/or contain structures resistant to extraction (Lienhard and Schäffer 2019). Daphnia contain a chitin carapace (Ebert 2005) interfering with extraction (Athanasio et al. 2016). *C. elegans* also has a protective collagen-like cuticle (Johnstone 1994). To combat these features, and the small body-size of both species, samples were pooled for extraction and RNA was quantified using a fluorescence-based method. RNA pooling has been used in microarray experiments where technical issues arise with low sample weights (Peng et al. 2003). The pooling of *C. elegans* and *D. magna* samples generates gene expression results that show trends which are not statistically significant results. Further replicates in future studies will compensate for the low body/tissue weights to increase RNA yield and avoid pooling.

To conclude, while the presence and implications of E2 in invertebrate organisms is unclear, the aim of this work was to determine whether it was bioavailable to the organisms, and once taken up, if it could subsequently cause a detrimental biological response. The uptake of SpECs has been confirmed by fluorescence microscopy for *D. magna* and *D. bugensis*. LCMSMS analysis also detected E2 variation among treatments for *C. elegans*, though no relationship to SpEC load was detected. A lack of E2 was detected in *D. bugensis* tissues suggests internal regulation. As an indicative biological response, the changes in gene expression of general stress biomarkers or specific to oestrogen chemicals were used. For *C. elegans*, the *NHR-14* gene suggests that SpECs modulate E2 impacts, but not general health responses (measured by *CAT*) (Fig. 3). For daphnia, SpECs alone and combined with E2 suggest an effect on *VTG1* and stress response (as *SOD*) (Fig. 3). For *D. bugensis* no significant difference in expression was detected for *ER* or *CAT* in response to E2 and/or SpECs treatment (Fig. 3) and either alternative contaminants, or biological endpoints, must be pursued to better investigate the dynamics of SpECs and bound chemicals in these species.
